# Mapping the knowledge structure and trends in Australian Indigenous health and wellbeing research from 2003 to 2022: a scientometric analysis

**DOI:** 10.3389/fsoc.2023.1290322

**Published:** 2023-11-30

**Authors:** Michelle A. Krahe, Kerry K. Hall, Peter J. Anderson, Cindy Shannon

**Affiliations:** ^1^Office of the Deputy Vice Chancellor (Indigenous, Diversity and Inclusion), Griffith University, Meadowbrook, QLD, Australia; ^2^College of Medicine and Dentistry, James Cook University, Cairns, QLD, Australia

**Keywords:** Aboriginal and Torres Strait Islander, Indigenous, Australia, health, wellbeing, scientometric analyses, trends, knowledge

## Abstract

The health and wellbeing of Australian Indigenous peoples is a nationally sanctioned priority, but despite this, few studies have comprehensively analyzed the features and characteristics of the research in the field. In this regard, a comprehensive scientometric analysis and knowledge mapping to systematically summarize and discuss the current state of research, research trends, and emerging areas of research were conducted. Original articles and reviews published between 2003 and 2022 were obtained from the Web of Science Core Collection. CiteSpace and VOSviewer software were used to perform scientometric analysis and knowledge mapping. An examination of document and citation trends, authors, institutions, countries/regions, journals, and keywords was untaken, while co-citation, co-occurrence, and burst analysis provide insights and future development in this area. A total of 2,468 documents in this field were retrieved. A gradual increase in the number of documents over the past two decades is observed, with the number of documents doubling every ~7.5 years. Author Thompson SC and Charles Darwin University published the most documents, and 85.6% were affiliated with only Australian-based researchers. The *Australian and New Zealand Journal of Public Health* is the most prominent journal publishing in the field. The most commonly co-occurring keyword was “health,” and the keyword “risk” had the longest citation burst. Five keyword clusters were identified; “cultural safety” was the largest. This study articulates the knowledge structure of the research, revealing a shift from population-level and data-driven studies to more applied research that informs Indigenous peoples health and wellbeing. Based on this review, we anticipate emergent research areas to (1) reflect a more comprehensive understanding of the multidimensional factors that shape Indigenous health and wellbeing; (2) move beyond a deficit-based perspective; (3) respect cultural protocols and protect the rights and privacy of Indigenous participants; (4) address racism and discrimination within the healthcare system; (5) foster respectful, equitable, and collaborative research practices with Indigenous peoples; (6) provide culturally appropriate and effective interventions for prevention, early intervention, and treatment; and (7) ensure equitable change in systems to enhance access, quality, and outcomes in health and wellbeing.

## 1 Introduction

Aboriginal and Torres Strait Islander peoples (herein Indigenous^1^), represent the oldest continuing cultures in the world. Since the colonization of Australia, Indigenous peoples have experienced profound trauma and losses in social and emotional, health, and wellbeing through the devastation or fragmentation of traditional lands, languages, culture and community (Dudgeon and Walker, 2015; Paradies, 2016). Today, Indigenous peoples account for 3.8% (an estimated 984,400 people) of the total Australian population and are projected to reach 1 million people by 2028 (Australian Bureau of Statistics, 2022). Despite steady improvements in the life outcomes of Indigenous peoples over the past few decades, a notable gap compared to the wider community remains (Australian Institute of Health and Welfare, 2015). In an unprecedented shift in the way governments have previously worked, closing this gap is a national priority that embraces the strength and resilience of Indigenous culture and communities as a foundation for partnership and shared decision-making (Arabena et al., 2014; Australian Government, 2021; National Indigenous Australians Agency, 2022).

Indigenous peoples view health as a holistic concept that encompasses more than just the absence of disease or illness. It embraces elements like cultural identity and spiritual well-being, family and kinship, connection to the land and its care, traditional knowledge and beliefs, language preservation, and active participation in community life, along with access to ancestral lands for both individuals and communities (AIHW, 2022). Indigenous leadership plays a crucial role in enhancing research impact and ensuring its benefits for Indigenous communities. This is achieved by prioritizing activities that hold significance and align with the community’s interests and cultural perspectives (Kiatkoski Kim et al., 2020). When research is led by Indigenous peoples, it has the potential to foster the creation of workforce development, strategies, policies, and procedures at regional, national, and global levels that genuinely support Indigenous peoples, all viewed through an Indigenous perspective.

Despite the previous reviews of Indigenous health research that have identified continued growth in outputs (Kinchin et al., 2017), lack of intervention research and research in urban settings (Jennings et al., 2021; McGuffog et al., 2023), and the need to hold to account health systems (Kennedy et al., 2022), there have been limited attempts to explore the evolution and current state of knowledge structure of the research. In 2006, Sanson-Fisher et al. (2006) examined the scientific literature related to the health of Indigenous peoples collectively from Australia, Canada, New Zealand and the United States at time points between 1987 and 2003. They conclude that the abundance of descriptive research is not considered an exemplar and encourage research organizations and researchers to consider this when developing research policies. In 2012, Derrick et al. (2012) published a bibliometric analysis of Indigenous health research in Australia (1972–2008). They conclude that while the volume of citations in selected health disciplines continues to grow, this still does not reflect the gravity of Indigenous health problems.

The National Health and Medical Research Council (NHMRC) has made significant commitments to Indigenous health and medical research in recent years (National Health and Medical Research Council, 2018). In 2018, they committed to allocate at least 5% of the Medical Research Endowment Account specifically to Indigenous health and medical research. The 2021 report demonstrates that this goal has been surpassed, evidenced by the funding of 206 active grants totaling over $58 million (National Health and Medical Research Council, 2021). However, despite more than 15 years since the introduction of the Australian Government’s Closing the Gap strategy, the 2031 targets are not progressing as planned. The 2022 Lowitja Institute Close the Gap Campaign Report (Lowitja Institute, 2022) underscores the importance of sustained investment in research that informs policy and practice reform as a critical step towards empowering Indigenous communities and improving health and social outcomes.

One direction is to investigate the evolution of a research topic. In our attempt to detect trends in Indigenous health and wellbeing research, we combine modelling and visualization to establish a knowledge base that will have important value for academics, practitioners, and government departments to formulate public health strategies and provide support and guidance for future research.

In this study, we combine bibliometric and scientometric techniques to analyze the knowledge structure regarding research productivity, and collaboration across authors, institutions, and countries/regions, and to reveal trends and forecast emerging areas of research. Despite similar approaches in other fields (You et al., 2021), this study is the first to detail a scientometric analysis of the characteristics, knowledge structure and trends in Indigenous health and wellbeing research. The key objectives of this study include:

*RQ*1. What are the trends and forecasted growth in documents?

*RQ*2. Who are the most influential authors, institutions, countries/regions, and journals?

*RQ*3. Which documents and keywords are the most impactful?

*RQ*4. What are the dominant topics, trends, and emerging research areas?

Addressing these research questions will fill important gaps in the current body of knowledge, and advance our understanding of research related to the health and wellbeing of Australian Indigenous peoples.

## 2 Materials and methods

### 2.1 Research design

This study examines the published scientific literature related to Indigenous health and wellbeing using scientometric analysis and knowledge mapping. The detailed procedure is discussed in the following sections.

### 2.2 Scientometric analysis

Scientometric analysis is a quantitative research method that focuses on the analysis and mapping of scientific literature, to explore research themes and collaboration clusters, and to identify gaps and trends (Mingers and Leydesdorff, 2015; López-Pernas et al., 2023). It involves the application of statistical and bibliometric techniques to evaluate and measure scientific activity to provide insights into the structure, growth, and impact of scientific knowledge (Noyons et al., 1999; Borgohain et al., 2021; Basumatary et al., 2022). Analyzing citation patterns, trends, and authorship networks, scientometric analysis can identify influential researchers, leading institutions, emerging research areas, and the overall development of scientific fields (Kastrin and Hristovski, 2021). In turn, this work can help researchers, policymakers, and institutions gain insights into the dynamics of scientific knowledge production, dissemination, and impact and it can inform decision-making related to resource allocation, funding strategies, identification of research trends, and evaluation of individual researchers, institutions, or research programs.

### 2.3 Search strategy and data collection

**Figure 1:**
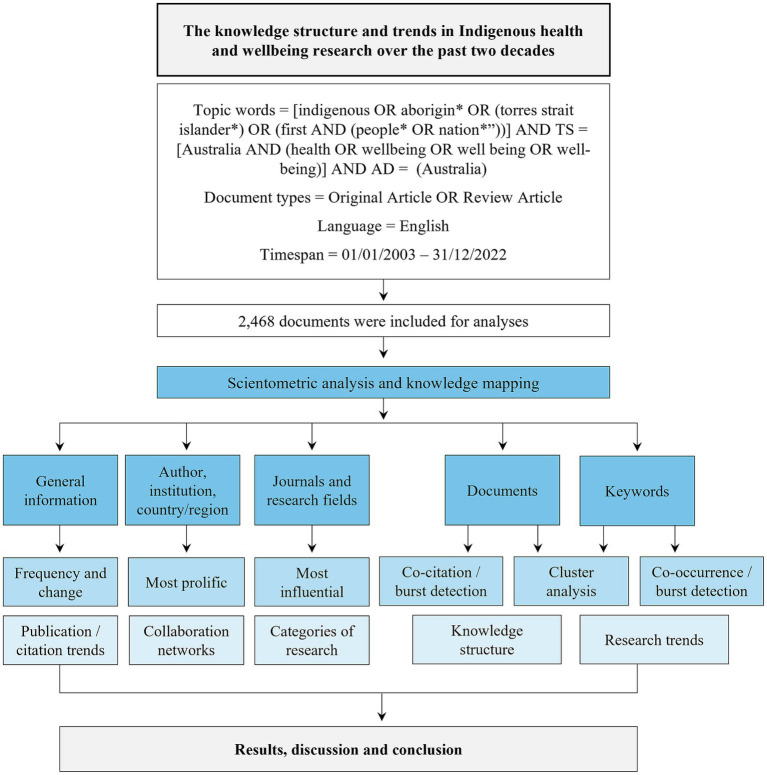
Methodological framework for the current study.

The selected source for the literature search and collection is the Web of Science Core Collection (WoSCC) database (Singh et al., 2021; Xia et al., 2021; Gusenbauer, 2022). A retrieval plan is detailed in Figure 1 for indexed documents relating to Indigenous health and wellbeing research, authored by researchers with an Australian affiliation, and published between 1 January 2003 and 31 December 2022. In order to mitigate the potential for bias resulting from ongoing database modifications, the retrieval and export of documents were executed on a single day (1 January 2023). We included papers that focused solely on Indigenous health and wellbeing, as well as those that incorporated data related to Indigenous health and wellbeing, such as the distribution of diseases or population-level risk factors. We adopted the Lowitja Institute search syntax for Aboriginal and Torres Strait Islander people (Lowitja Institute, 2022) and the final search string applied in the WoSCC: (TI = [Indigenous OR Aborigin* OR Torres Strait Islander* OR First AND (People* OR Nation*”)] AND TS = [Australia AND (health OR wellbeing OR wellbeing OR well-being)] AND AD = [Australia] AND DOP = [01-Jan-2003 to 31-Dec-2022] AND Language = [English]).

Original articles and reviews (including early access articles) were included. A total of 2,800 documents were identified, of which, 332 records were excluded: meeting abstracts, editorial material, letters, and proceeding/data papers. Ethical approval was not applicable. For the present study, 2,468 documents (2,198 original articles and 279 reviews) were obtained as the final dataset for analysis. Data were obtained from WoSCC in .csv format for analysis, and in plain text format (full records and cited references) to visualize the data.

### 2.4 Measures

- Number of documents: To quantify the amount of research conducted over time, as distributed by authors, institutions, countries/regions, journals, and research categories.
- Number of citations: To examine trends in research over time, and to identify the most influential authors, institutions, countries/regions, and journals.
- Co-authorship: To evaluate collaboration networks of authors, institutions, and countries/regions, providing insight into the social connections across the domain.
- Co-citation: To analyze the frequency with which documents are cited together by other researchers, and to reveal clusters of interdisciplinary research trends.
- Co-occurrence: To identify related keywords and measure the strength of their links to visualize trending and emerging research themes.
- h-index: To quantify both productivity and impact.
- Journal Impact factor (JIF) and quartile in category (Q1-Q4) were obtained from the 2021 Journal Citation Report (Clarivate, 2022).

### 2.5 Data analysis and visualization

Based on the analysis approach, Microsoft Excel 2022 (Redmond, WA, United States) was used to analyze and graph document and citation metrics. The most influential documents were identified based on their citation count, and the top 10 authors, institutions, countries/regions, and journals were identified based on the number of documents. Journal research categories are presented as document counts and proportions.

VOSviewer (version 1.6.16) (Leiden University, Leiden, the Netherlands) was used to visualize the networks of authors and institutions (Fonseca Bde et al., 2016). Full-counting was applied and the threshold was set to ≥5 co-authored documents (Egghe et al., 2000). Based on these settings, the number of documents, citations, and total link strength (TLS) were determined. In this analysis, the nodes represent the author or institution, the size of the node represents the number of documents, and the lines between nodes represent co-authorship links. The thickness of the line depicts link strength. Clustering analysis was used to identify sub-clusters of collaboration from the overall structure of the literature (Rodriguez and Laio, 2014; Chen, 2017; Jiang et al., 2017; Singh et al., 2021).

CiteSpace (version 6.1.R6) (Wang and Lu, 2019) was used to explore the knowledge structure and research trends in the scientific literature. Parameters for this investigation were set to: time-slicing from 2003 to 2022 (4 years per slice), look back years = −1, link retaining factor = −1, top N% = 100%, top N = 50, and g-index = 25. Default settings for text processing and links were preserved and metrics such as citation burstiness, Sigma, Silhouette and betweenness centrality were reported.

To achieve this, the following scientometric techniques were employed:

- Co-citation analysis of documents quantifies how often documents are cited together, revealing influential publications with high citations (bursts) and related clusters.
- Keyword co-occurrence analysis identifies the most important keywords (extracted after searching the titles, abstract, and keywords) that represent the conceptual building blocks of the scientific literature.
- Burst detection of highly co-cited documents and keyword co-occurrences highlight significant literature and keywords (Su and Lee, 2010). The burst strength list is created using an algorithm mapping of hierarchical structure to capture increases in popularity within a specified period
- Cluster analysis divides the networks into clusters by extricating terms from the title, abstract, author keywords, and keyword plus using the default algorithm log-likelihood ratio test. The top 50 keywords that appear in each time slice are presented and clusters are identified using distinct colors and labelled using CiteSpace.
- Timeline visualizations are generated from cluster analysis on a discrete horizontal axis. The term source includes title, abstract, author, keywords, and keyword plus of cited documents using the log-likelihood ratio algorithm. Clusters are arranged in a vertical manner descending in size, with the largest cluster at the top (#0). Each node represents a document, links between two nodes represent the co-citation/co-occurrence, and color corresponds to the year they most recently appeared. Documents with a citation burst and/or highly cited are denoted with a bright purple ring.

## 3 Results

### 3.1 Trends in publications and citations

**Figure 2:**
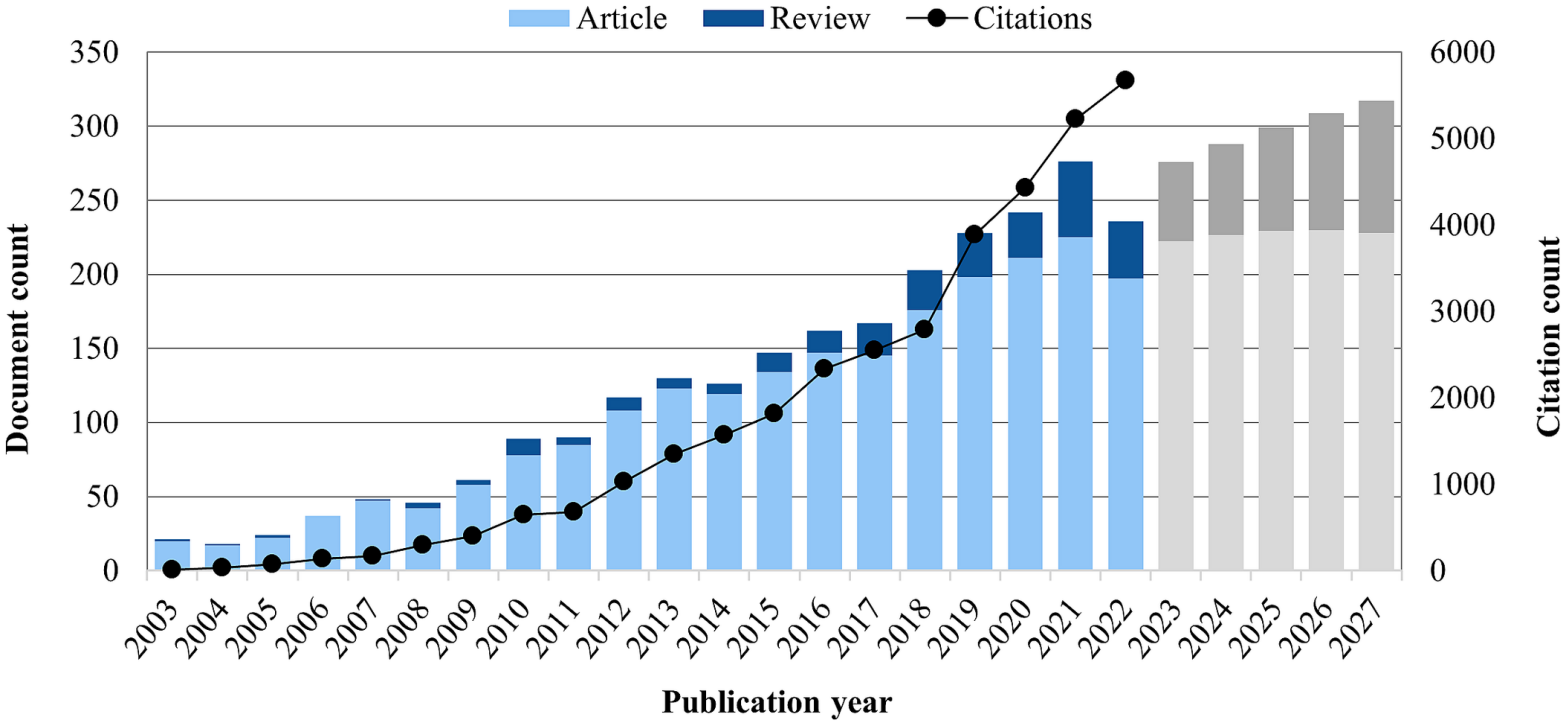
Document counts and citation growth trends of Indigenous health and wellbeing research (2003–2022), and document growth forecast for the next 5 years (2023–2027).

Overall, publications have steadily increased from 21 documents in 2003, to 236 documents in 2022 (an average of 123 documents published/year). The average annual performance is 15.1% (95% CI 8.1 to 23.1%) and on average, the number of documents doubled every 7.5 years (Figure 2). In the first decade, the average annual performance of 23.2% (95% CI 13.7 to 36.9%) indicates an initial rapid growth period, which slowed to a stable 7.8% (95% CI 6.1 to 13.9%) in the second decade. A regression exponential trend line indicates consistent growth (*R*^2^ = 0.9792) over time. A forecast estimate of future trends in Indigenous health and wellbeing research based on the equation of model fit indicates an additional 1,488 documents (1,136 original articles and 353 reviews) published in the next five years (2023–2027); representing an average annual performance of 6.20% (95% CI 4.7 to 10.9%).

### 3.2 Analysis of authors

The author network for Indigenous health and wellbeing research consists of 6,580 authors; 95% (*n* = 2,344) of documents are co-authored, and the median number of authors per paper is five (range: 1 to 65). The top 10 authors with the greatest number of documents are listed in Table 1 and collectively account for almost one quarter (*n* = 608; 24.6%) of all documents included in this study. These authors are affiliated with eight institutions, four of which are members of Australia’s leading research-intensive universities, known as the Group of Eight (Go8) (The Group of Eight Ltd, 2022). Thompson, SC has the greatest number of documents (*n* = 87), total citations (*n* = 1,749), and *h*-index (Borgohain et al., 2021) related to the dataset in this study.

**Table 1:** Top 10 authors publishing Indigenous health and wellbeing research (2003–2022).

#	Author	No. of documents^a^	Total citations (average/document)^a^	*h*-index^a^
1	Thompson SC	87	1,749 (20.10)	24
2	Brown A	74	1,130 (15.27)	19
3	Jamieson LM	69	668 (9.68)	16
4	Eades S	65	940 (14.46)	18
5	Bailie R	61	1,203 (19.72)	19
6	Ward J	53	484 (9.13)	13
7	Garvey G	51	538 (10.55)	13
8	Clough A	40	671 (16.78)	16
9	Atkinson D	38	427 (10.95)	12
10	O’Dea K	36	761 (21.14)	16

a Metrics reported are calculated for the dataset included in this study only.

**Figure 3:**
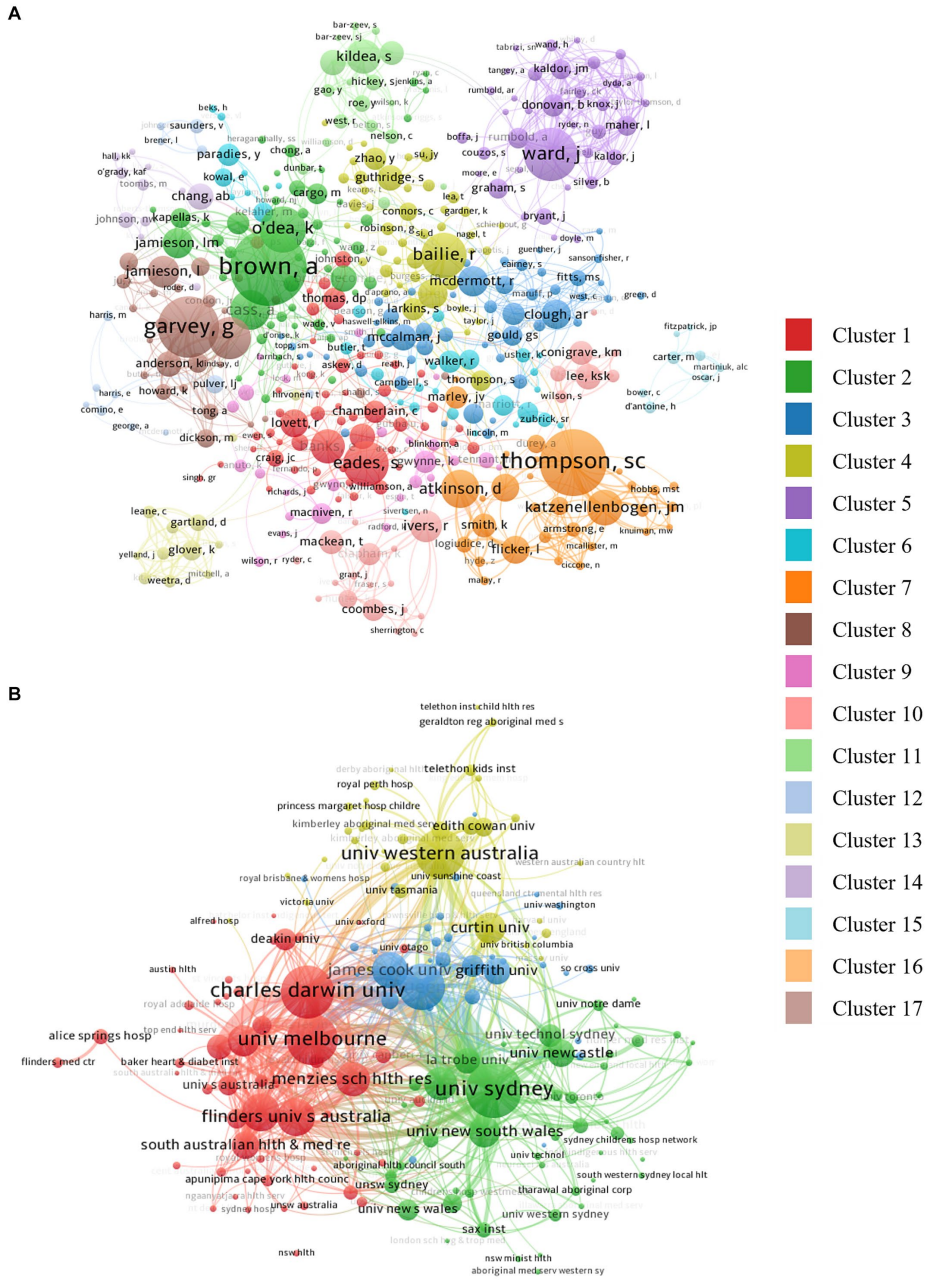
Visualization of the **(A)** authorship collaboration network, and **(B)** institutions co-authorship.

Figure 3A presents a visualization of the co-authorship network among 446 authors, grouped into 17 clusters with 3,197 links. In this analysis, Brown, A (cluster 2, 51 nodes) has the greatest number of co-authored documents (*n* = 57 and 79 links), Thompson, SC (cluster 7, 31 nodes) has the most citations (*n* = 931), and Ward, J (cluster 5, 36 nodes) has the highest TLS of 209. Other authors with notably high TLS include Brown, A (196), Garvey, G (cluster 8, 27 nodes) (179), and Thompson SC (157).

### 3.3 Analysis of institutions

A network of 1,616 institutions has contributed to the research; Table 2 lists the top 10 institutions. Of note, six of these institutions are members of Australia’s Go8 (The Group of Eight Ltd, 2022) universities and one, the Menzies School of Health Research, is a young (38 years) medical health research institute dedicated to improving health outcomes for Indigenous Australians. Charles Darwin University ranked first with 508 articles followed by the University of Sydney (*n* = 476), and the Menzies School of Health Research (*n* = 417). A visualization of the institution network highlights four clusters. As can be seen in Figure 3B, cluster 1 (red, 56 nodes) is co-led by the University of Melbourne (links = 129, TLS = 799) and Charles Darwin University (links = 107, TLS = 775). Cluster 2 (green, 49 nodes) is led by the University of Sydney (TLS = 1,058), cluster 3 (blue, 33 nodes) is led by The University of Queensland (TLS = 720), and cluster 4 (yellow, 32 nodes) is led by the University of Western Australia (TLS = 641). Other institutions with high TLS include Flinders University (490), Menzies School of Health Research (481), and the University of Adelaide (473).

**Table 2:** Top 10 institutions publishing Indigenous health and wellbeing research (2003–2022).

#	Institution	Location	Established	No. of documents^a^	Total citations (average/document)^a^
1	Charles Darwin University^b^	Northern Territory	2003	508	8,248 (16.11)
2	University of Sydney	New South Wales	1850	476	6,366 (13.32)
3	Menzies School of Health Research^c^	Northern Territory	1985	417	7,041 (16.72)
4	University of Melbourne	Victoria	1853	357	6,147 (17.17)
5	University of Western Australia	Western Australia	1911	354	5,441 (15.33)
6	University of Queensland	Queensland	1909	312	4,499 (14.42)
7	University of New South Wales	New South Wales	1949	302	3,338 (11.05)
8	Flinders University	South Australia	1966	237	2,697 (11.38)
9	University of Adelaide	South Australia	1874	215	2,593 (12.06)
10	James Cook University	Queensland	1970	206	3,341 (16.22)

a Metrics reported are calculated for the dataset included in this study only.

b Established after a merger between three institutions.

c An independent medical and research institute within Charles Darwin University.

### 3.4 Analysis of countries/regions

A total of 54 countries/regions have contributed to the production of Indigenous health and wellbeing research over the past two decades; 85.6% (*n* = 2,113) are authored by Australian institutions only, and the remaining 355 publications are affiliated with authors predominantly from the United States (25.07%), Canada (24.22%), England (22.53%), and New Zealand (20.28%) (Table 3). While the number of documents co-authored with the People’s Republic of China and Brazil is not high, the average citation rate is outstanding, suggesting that the quality and application of these publications are elevated.

**Table 3:** Top 10 countries/regions contributing to Indigenous health and wellbeing research (2003–2022).

#	Country/Region	No. of documents^a^	Total citations (average/document)^a^
1	USA	89	2,239 (24.88)
2	Canada	86	1,859 (21.62)
3	England	80	1,148 (14.17)
4	New Zealand	72	2,012 (27.56)
5	Scotland	12	199 (16.58)
6	People’s Republic China	11	660 (55.00)
7	Brazil	10	626 (62.60)
8	South Africa	6	75 (12.50)
9	Germany	6	58 (9.67)
10	France	5	155 (31.00)

a Metrics reported are calculated for the dataset included in this study only.

### 3.5 Analysis of journals and research fields

Over the past two decades, a total of 632 journals have been the 2,468 documents. The top 10 most active journals are presented in Table 4 and account for 30% (n = 739) of all documents. The *Australian and New Zealand Journal of Public Health* has published the most documents (n = 113), followed by *the International Journal of Environmental Research and Public Health* (n = 96). The *Medical Journal of Australia* is the only Q1-ranked journal and has the highest impact factor at 12.766. Six of the 10 journals are classified as *Public, Environmental, and Occupational Health* based on the journal’s research area (as categorized by WoSCC).

**Table 4:** Top 10 journals publishing Indigenous health and wellbeing research (2003–2022).

#	Journal	No. of documents^a^	Total citations (average/document)^a^	Category (Q1-Q4)^a^	JIF^a^
1	Australian and New Zealand Journal of Public Health	113	1,735 (15.65)	Public, environmental, and occupational health (Q2)	3.755
2	International Journal of Environmental Research and Public Health	96	522 (6.87)	Environmental sciences (Q2); Public, environmental, and occupational health (Q2)	4.614
3	BMC Public Health	89	1,285 (14.44)	Public, environmental, and occupational health (Q2)	4.135
4	Medical Journal of Australia	82	3,251 (35.40)	Medicine, general and internal (Q1)	12.776
5	BMC Health Services Research	78	1,405 (18.01)	Health care sciences and services (Q3)	2.908
6	BMJ Open	75	674 (10.21)	Medicine, general and internal (Q2)	3.006
7	Rural and Remote Health	56	570 (10.96)	Public, environmental, and occupational health (Q3)	2.733
8	Australian Journal of Primary Health	53	491 (9.44)	Health care sciences and services (Q4); Primary healthcare (Q4); Public, environmental, and occupational health (Q4)	1.72
9	Australian Health Review	49	621 (13.50)	Health care sciences and services (Q4)	1.837
10	Australian Journal of Rural Health	46	582 (14.20)	Nursing (Q3); Public, environmental, and occupational health (Q3)	2.606

a Metrics reported are calculated for the dataset included in this study only.

JIF, Journal Impact Factor according to Journal Citation Reports 2021; Q1–Q4 = quartile in category ranking by JIF.

**Figure 4:**
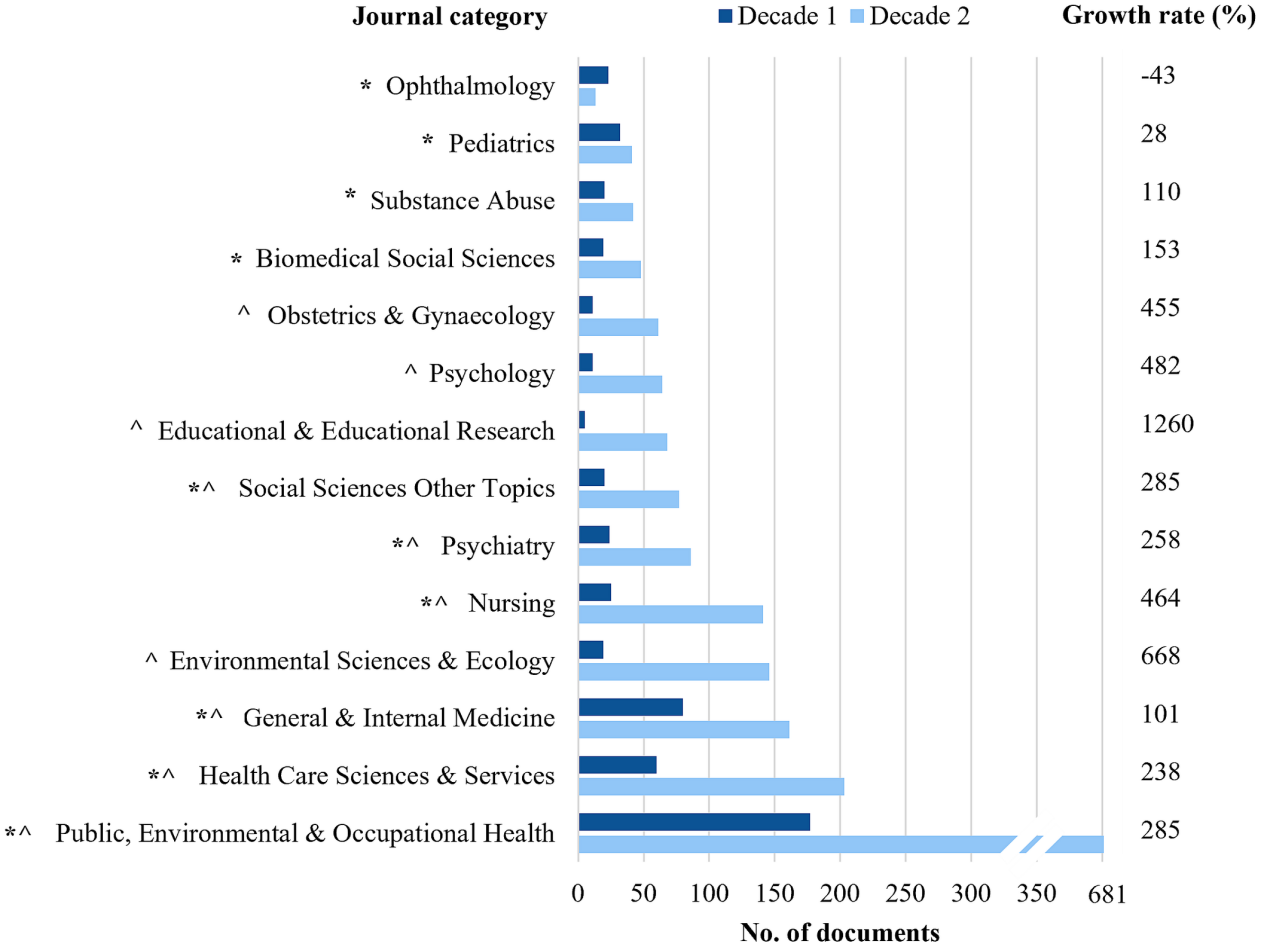
Top 10 journal research categories for documents published between 2003 to 2012 (decade 1) and 2013 to 2022 (decade 2). Growth rate (%) indicates the change in the number of publications between decade 1 and decade 2. *categories included in the top 10 for decade 1; ^categories included in the top 10 for decade 2.

Figure 4 compares the top 10 journal categories for documents published in the first (2003–2012) and second (2013–2022) decades. Of note, six categories remain in the top 10, with “public, environment and occupational health,” “healthcare sciences and services,” and “general and internal medicine” as the top three categories. Documents published in journals categorized as “public, environmental and occupational health” had the greatest increase in the number of publications from 177 in decade 1 to 681 in decade 2, and as a proportion, publications categorized as “education and educational research” increased by 1,260%. Despite the categories of “substance abuse,” and “biomedical social sciences,” not being included in the top 10 for decade 2, documents still increased by 2–2.5 times. “Ophthalmology” is the only category to decrease between decades.

### 3.6 Analysis of documents

**Figure 5:**
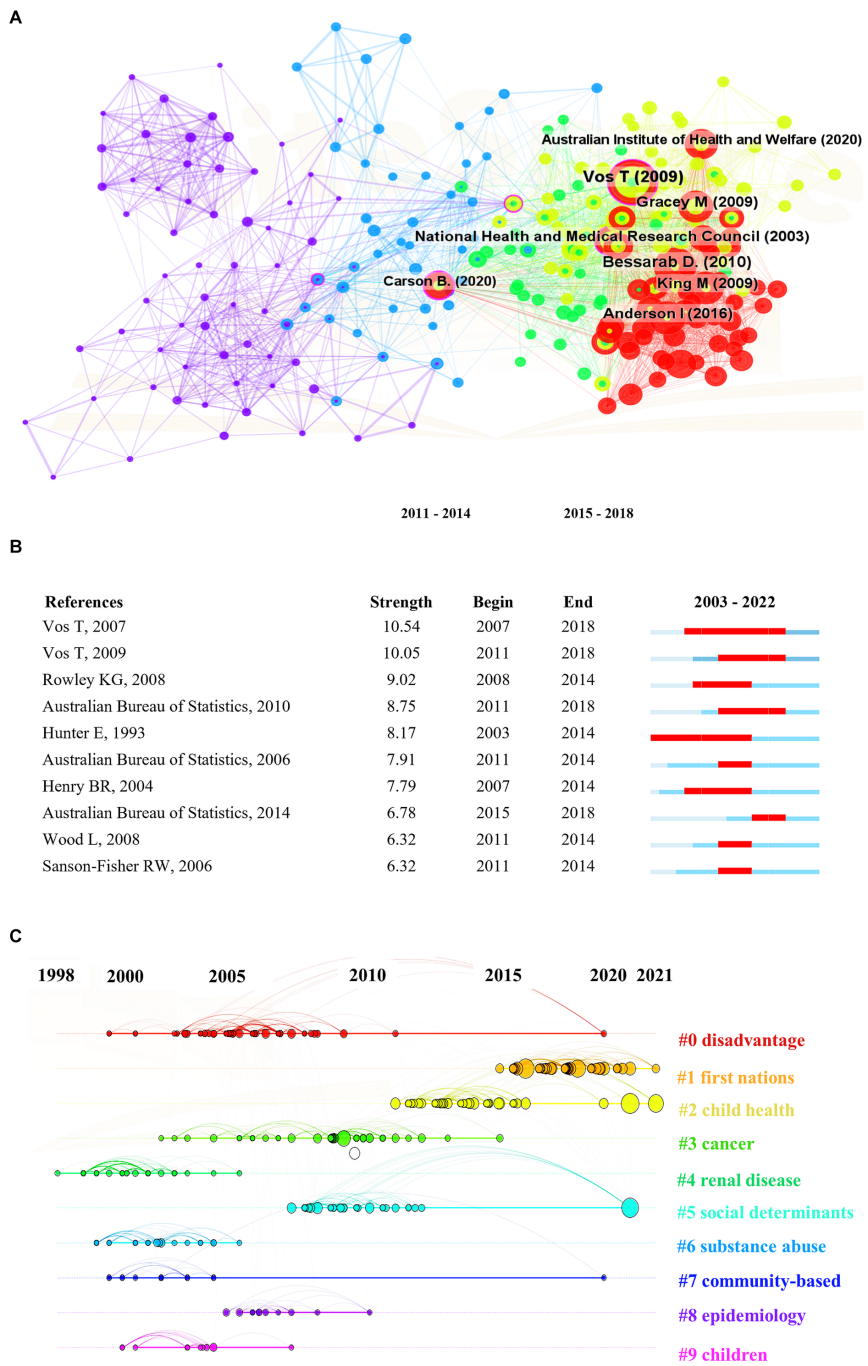
Visualization of the **(A)** document co-citation network, **(B)** top 10 documents with the strongest citation bursts, and **(C)** document co-citation clusters over time.

Key steps to building a knowledge structure include identification of highly cited articles and key research areas. Figure 5A presents a visualization of the network of highly co-cited documents (*n* = 241, links = 2,987). To ensure a clearer picture, only nodes with ≥60 co-citations are labelled. The top three highly co-cited documents are the publications by Gracey and King (2009), Vos et al. (2009), and Bessarab and Ng'andu (2010). The publication by Carson et al. (2007), titled “social determinants of Indigenous health,” examines the enduring health impacts of the Indigenous experience of dispossession, colonial rule, and racism. Published in 2007, it stands out as an influential document in the literature network (centrality score of 0.60). Published in earlier years, documents by King et al. (2009), and the NHMRC (National Health and Medical Research Council, 2018) continue to be consistently referenced and cited extensively in recent times.

Figure 5B shows the top 10 co-cited documents with the strongest citation bursts. Time is represented by the blue line, and the period when the document bust occurred, by the red line. The article with the longest citation burst lasting from 2007 to 2018 is titled “burden of disease and injury in Aboriginal and Torres Strait Islander peoples: the Indigenous health gap” (Vos et al., 2009). This study uses national population health datasets and Indigenous-specific epidemiological studies to measure the Indigenous health gap.

Based on the co-citation analysis network (Figure 5A), cluster analysis identified 10 research clusters (Trujillo and Long, 2018) which are visualized over time in Figure 5C (detailed in Supplementary File 1). The Q Score and S value (0.7965 and 0.9198, respectively) indicate that the network is reasonably divided, and the precision of clustering is high. The ranking of clusters is determined by the number of documents, where “disadvantage” (#0) is the largest cluster and “children” (#9) is the smallest. The clusters of “First Nations” (#1), “child health” (#2), and “social determinants” (#5) are trends in Indigenous health and wellbeing research in recent years.

### 3.7 Analysis of keywords

**Figure 6:**
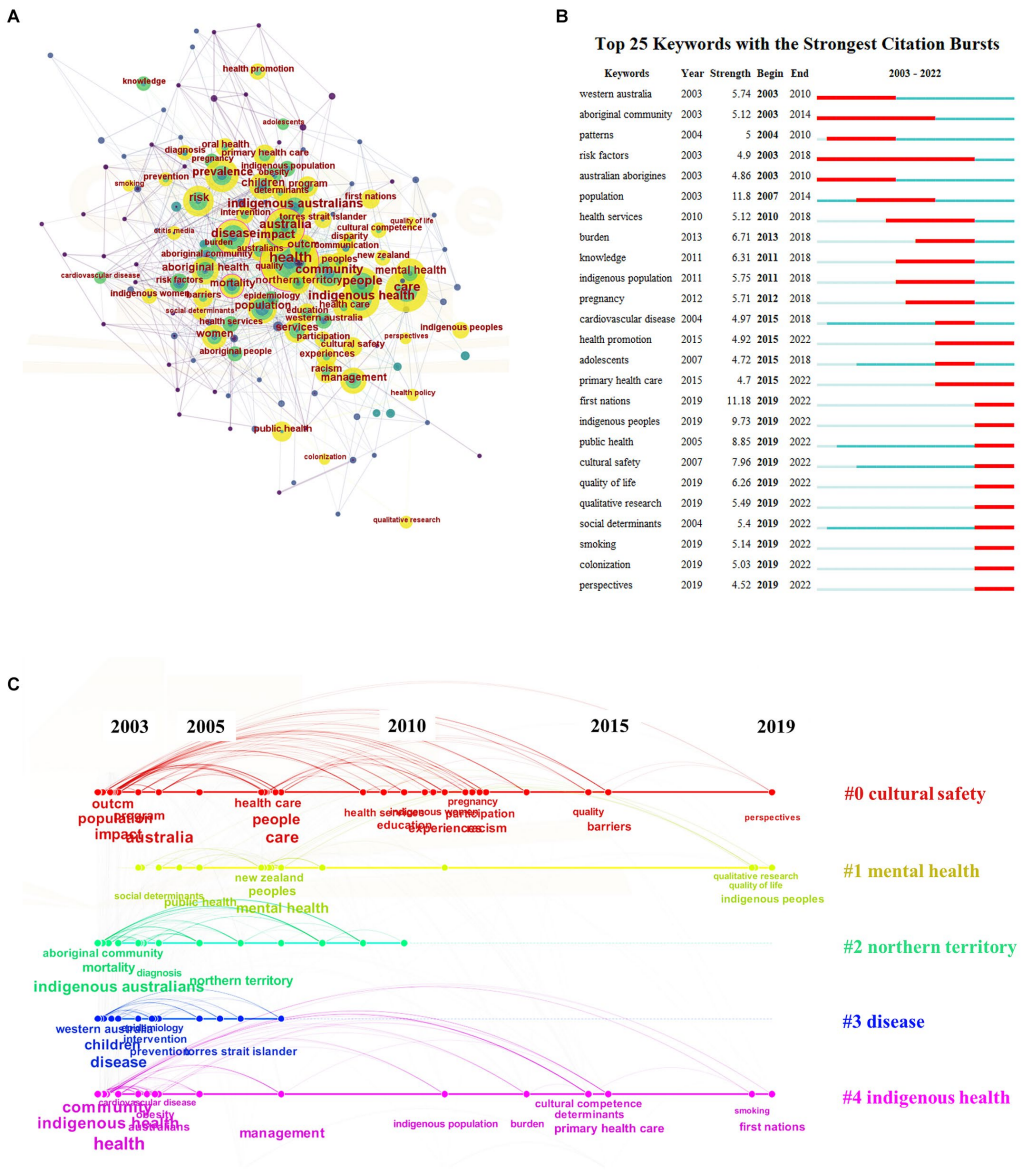
Visualization of the **(A)** keyword co-occurrence network, **(B)** keywords with the strongest citation bursts, and **(C)** keyword co-occurrence clusters over time. Year = is the earliest year of all publications being analyzed; strength = is an indicator related to the frequency of the keyword in a short time; begin and end = refers to the year of beginning and ending of the emergence of the keywords.

The keywords of a document reflect the research focus of publications. Here, we analyze the co-occurrence of keyword trends to explore research topics and frontiers in the field of Indigenous health and wellbeing. A network of 145 nodes (related keywords), and 862 links (connections), with a density of 0.0826 is shown in Figure 6A. The most commonly co-occurring keywords are “health” (363 times), “care” (187 times), “Australia” (184 times), “Indigenous health” (168 times), and “community” (166 times). Burst detection identified 42 keywords with a minimum duration of two years; the 25 keywords with the strongest citation bursts are shown in Figure 6B. The term “risk factors” had the longest burst period (2003–2015) and keyword bursts lasting until 2022 include: “Indigenous peoples,” “cultural safety,” “qualitative research,” “social determinants,” “smoking,” “perspectives,” “First Nations,” “public health,” “quality of life,” and “colonization.” These 10 keywords reflect the most recent research trends and belong to clusters labelled “cultural safety,” “mental health” and “Indigenous health.”

The keyword network was divided into five clusters which represent the main keyword categories of Indigenous health and wellbeing research (see Supplementary File 2). The evolution of keywords over time can be seen in Figure 6C. The largest cluster (#0) is “cultural safety” with 35 keywords related to cultural safety in healthcare, disparities, racism, barriers to participation, education, communication, quality of health services, women’s health, and Indigenous populations. Cluster two is “mental health” (#1) with 29 keywords. The theme suggests an exploration of mental health policies, interventions, and research aimed at improving mental wellbeing and addressing the unique challenges faced by Indigenous peoples. The third cluster (#2) “Northern Territory” contains 29 topics, that together suggest an exploration of the patterns, diagnoses, and associations related to prominent health issues within Indigenous communities. The fourth cluster (#3) is “disease” with 22 topics that highlight various aspects related to disease, particularly in children, in the context of a place (Western Australia). The final cluster “Indigenous health” (#4) has 22 topics that cover primary healthcare, risk, prevalence, and determinants of health.

## 4 Discussion

This study provides an extended view of Indigenous health and wellbeing research published over the past two decades – offering insights into the achievements, knowledge structure, research trends and emerging areas. It is the first to apply scientometric techniques to represent the dynamic and structural features of the research visually.

Our analysis of 2,468 documents reveals prominent authors, institutions, countries/regions, and journals who have contributed to Indigenous health and wellbeing research over the past two decades - with an estimated 60% increase in the volume of documents by 2027. This growth likely reflects the growing national priorities for the health and wellbeing outcomes for Indigenous peoples and communities in Australia (National Health and Medical Research Council, 2018; Lowitja Institute, 2022), the availability of guidelines and frameworks related to the ethical conduct of research with Indigenous peoples and communities (National Health and Medical Research Council, 2018; Australian Institute of Aboriginal and Torres Strait Islander Studies, 2020), along with the significant commitment in targeted research funding (National Health and Medical Research Council, 2018). In particular, the NHMRC has committed 5% of its total research funding budget to Indigenous health and medical research since 2008; in 2021 the NHMRC allocated 7.09% of funding or $58.1 million (National Health and Medical Research Council, 2018).

Each of the top 10 authors published at least 36 documents. Thompson SC had the greatest number of documents, number of total citations, and highest h-index. The focus of Professor Thompson’s research is the prevention and management of chronic disease in remote communities and Aboriginal health. In a 2010 review of Australia’s National bowel cancer screening program, barriers that exclude vulnerable minorities, including Indigenous groups, from participating in bowel cancer screening initiatives and the greater incidence of late-stage cancer and mortality among Indigenous peoples are highlighted (Christou et al., 2010). Since then, studies to address barriers through appropriate health promotion and education have been rolled out. This includes the National Indigenous Bowel Screening pilot among 44 Indigenous primary healthcare centers (Menzies School of Health Research, 2020) in 2018 which is now available nationally.

According to our analysis, authors affiliated with Charles Darwin University had published the most documents (*n* = 508; 20.6%), and despite the University of Melbourne ranking fourth, it had the highest average citation rate of 17.7. The University of Sydney co-authored the greatest number of publications with international institutions (*n* = 84). It is worth noting that these lead institutions are renowned research and higher education organizations with prominent positions in Indigenous health research portfolios. Collectively, authors with affiliations to institutions based in the USA co-authored the most documents with Australia, albeit authors affiliated with the University of Toronto had the greatest single number of documents (*n* = 31).

The *Australian and New Zealand Journal of Public Health* published the largest number of documents (*n* = 113), and the *Medical Journal of Australia* had the highest impact factor (12.776) and citation count (*n* = 3,251). It is worth noting that most JCR partitions are categorized as Q2 and only one is a Q1 journal. These data will help researchers when they submit articles about Indigenous health and wellbeing in the future.

### 4.1 Knowledge structure

Central to the influence of relevant literature on the topic is the number of citations documents have received. In this study, 181 papers received only one citation, and 270 have not yet been cited. The average number of citations per document is 14.19 with a h-index of 68. Among the top 10 documents with the greatest citation rate (Supplementary File 3), nine of these papers are original articles and one is a review. Three are published in the *Medical Journal of Australia* (IF = 7.738) and two in the *Lancet* (IF = 79.321). The document that received the most citations (*n* = 481) accounts for 1.37% of the total citation count (Anderson et al., 2016) and is also recognized as a highly cited paper in the field of clinical medicine. Titled “Indigenous and tribal peoples’ health (The Lancet-Lowitja Institute Global Collaboration): a population study,” it is a large population study that reviews the health and social outcomes for Aboriginal and Torres Strait Islander peoples from across 23 countries. It is noteworthy that this article containing 65 authors and 43 affiliations, had one of the longest citation burst values lasting from 2019 to 2022. Reading the 10 most influential documents can assist researchers in gaining a foundational understanding of the knowledge structure pertaining to Indigenous health and wellbeing research.

The dynamic structure of research in this field is characteristic of documents with strong citation bursts and co-citation clusters. Statistics from CiteSpace identified that 44 articles broke out in recent years (2019–2022), of which 42 belonged to cluster #1 “First Nations” and two belonged to cluster #2 “child health” (Figure 5C). The “First Nations” cluster is concentrated between 2015 to 2022. Central to this cluster are papers that explore strengths-based approaches to Indigenous health. These include the paper by Askew et al. (2020) “Closing the gap between rhetoric and practice in strengths-based approaches to Indigenous public health: a qualitative study,” Harfield et al. (2020) “Assessing the quality of health research from an Indigenous perspective: the Aboriginal and Torres Strait Islander quality appraisal tool,” Paradies (2016) “Colonization, racism and indigenous health.” Child health is another hotspot that presents in 2011 in the highly co-cited editorial titled “Social determinants and the health of Indigenous Australians” (Marmot, 2011). In this short paper, Professor Marmot discusses the large social inequalities and the 17-year age gap in life expectancy between Indigenous and non-Indigenous Australians. The influence of early childhood development is discussed in relation to access to education suggesting that the environment in early childhood is key to health status along the social gradient (Marmot et al., 2008). A central paper within this cluster is also the highly co-cited systematic review of interventions for Indigenous peoples with chronic diseases by Gibson et al. (2015).

### 4.2 Research trends

Next, we reflect on trends in the research based on the timeline view of keyword co-occurrence clusters (Figure 6C). In the early 2000s, Indigenous health and wellbeing research had a particular focus on the prevalence, impact, and risk factors associated with various chronic diseases, including mental health, infectious diseases, cardiovascular disease, obesity, renal disease, otitis media, and oral health. This early research sought to understand the social determinants influencing health outcomes, such as cultural safety, access to healthcare services, and the impact of disparities in healthcare provision. The research tended to explore patterns of disease prevalence and mortality rates among Indigenous populations, including children, women, adolescents, and infants, in efforts to develop preventive programs and effective management strategies for Indigenous peoples and communities, particularly in regions such as the Northern Territory and Western Australia. Ultimately, the research sought to articulate health disparities and outcomes from intervention studies in Indigenous communities.

During the mid-2000s, Indigenous health and wellbeing research shifted to understanding and improving the experiences and participation of Indigenous peoples in health services. It explored the role of communication and cultural knowledge in enhancing healthcare interactions and outcomes and it sought to identify the unique challenges faced by Indigenous women in accessing and receiving appropriate healthcare services. The impact of cultural factors on health-seeking behaviors, and the role of education and knowledge exchange in improving health literacy within Indigenous communities is also a focus. This research would inform strategies to improve healthcare delivery, promote culturally sensitive practices, and develop targeted interventions that address the specific needs and experiences of Indigenous peoples. Ultimately, the research sought to contribute by ensuring active participation and improved experiences in healthcare settings for Indigenous communities.

By the late 2000s, a surge in research to understand and mitigate the burden experienced by Indigenous peoples in Australia, particularly associated with the impact of racism, historical colonization, and socio-cultural determinants, can be observed. Although instances of racism in Aboriginal and Torres Strait Islander health have been documented since the introduction of Closing the Gap in 2020, it was the Black Lives Matter movement that garnered worldwide recognition (The Lancet, 2020). Studies addressing barriers that hinder Indigenous peoples’ access to quality primary healthcare services and exploring strategies that promote cultural competence are trending. The research emphasizes the importance of health promotion and physical activity in improving the quality of life and endorses the perspectives and experiences of Indigenous peoples to gain a deeper understanding of their needs and challenges. Inevitably, this research is designed to inform health policies and initiatives aimed at reducing health disparities and addressing the impact of racism on health and wellbeing (Gatwiri et al., 2021). Unless the historical and contemporary determinants of Indigenous health and wellbeing are addressed, the development of a culturally appropriate and equitable healthcare system is ambitious.

### 4.3 Emergent research areas

**Figure 7:**
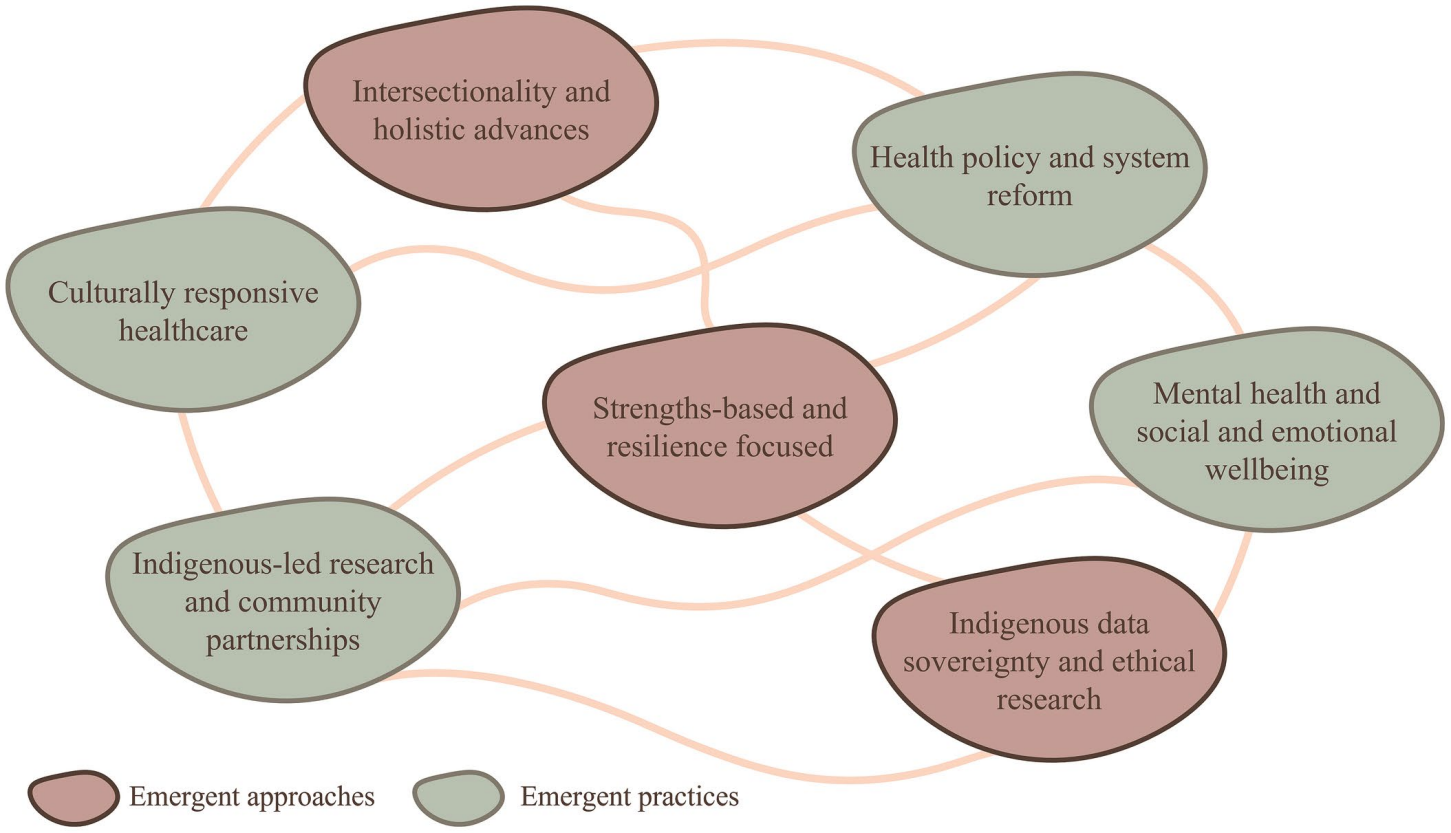
Emergent areas of research in Indigenous health and wellbeing research, as distributed by interconnected research approaches and research practices.

Despite the high level of investment, the gap in health and wellbeing outcomes between Indigenous and non-indigenous Australians remains alarmingly wide. Based on this study and through extensive reading of the literature, we consider the following areas to be emergent research approaches and practices (Figure 7).

Emergent approaches will contribute to a more holistic understanding of health and wellbeing, foster respect for Indigenous ways of knowing, and promote more equitable and inclusive research practices. An example is the concept of Two-Eyed Seeing introduced by Mi’kmaq Elders Albert and Murdena Marshall (Whiting et al., 2018). This approach seeks to integrate both Indigenous knowledge and Western scientific knowledge to address health disparities and promote wellbeing. It would involve combining traditional healing practices, cultural beliefs, and community wisdom with evidence-based medical and scientific approaches. Respecting the inherent strengths and knowledge present within Indigenous communities, while also valuing the advancements of Western medicine and research may include approaches such as:

- Intersectionality and holistic advances: Taking a more comprehensive understanding of the complex and multidimensional factors that shape Indigenous health, but in particular, wellbeing. This includes exploring the intersectionality of various factors, such as gender, age, socioeconomic status, highest educational attainment, and geographic location, to gain a more comprehensive and nuanced understanding of disparities, and lead to more effective interventions and policies that address the diverse needs and experiences of Indigenous peoples, recognizing their cultural strengths and promoting holistic health outcomes.
- Strengths-based and resilience-focused: Exploring the strengths and resilience within Indigenous communities, highlighting protective factors and successful health promotion strategies. This includes Indigenous leadership and governance in decision-making processes, policy development, and program implementation, or approaches that recognize the wisdom and guidance of Elders and traditional knowledge holders within communities. This approach aims to move beyond a deficit-based perspective, promote culturally appropriate and sustainable strategies, and empower Indigenous peoples to take an active role in their health and wellbeing.
- Indigenous data sovereignty and ethical research: A growing emphasis on Indigenous data sovereignty, which involves Indigenous control and ownership of data collected from the community. Researchers need to uphold ethical research practices that respect cultural protocols and protect the rights and privacy of Indigenous participants.

Emergent practices signify a shift toward more respectful, equitable, and effective research practices that honor Indigenous self-determination and prioritize the wellbeing of Indigenous peoples. An example is community-based participatory research that engages Indigenous communities as active partners in the research process, from defining research questions to interpreting results and implementing findings. This approach aims to address the historical power imbalances that have often characterized research involving Indigenous peoples and ensure that research is conducted in a way that aligns with the values, needs, and priorities of the community. Practices may include:

- Culturally responsive healthcare: Delving deeper into the concept of cultural safety in healthcare settings, examining strategies and interventions that promote culturally competent care delivery and addressing racism and discrimination within the healthcare system are warranted. This includes cultural awareness and competence training, communication collaborative decision-making, respect for cultural protocols and practices, workforce development, and addressing systemic issues and power imbalance.
- Indigenous-led research and community partnerships: There is increasing recognition of the importance of Indigenous-led research and community partnerships that foster respectful, equitable, and collaborative research practices and value Indigenous knowledge, perspectives, and self-determination. Research that involves close collaborations with Indigenous communities, ensuring their active participation in research design, implementation, and decision-making processes.
- Mental health and social and emotional wellbeing: While already a focus in earlier research, a deeper understanding of the specific mental health challenges faced by Indigenous peoples is culturally appropriate and culturally safe for prevention, early intervention, and treatment.
- Health policy and system reform: Continuing to inform health policies, and organizational and administrative procedures that advocate for system changes to reduce health disparities, improve access to quality healthcare services, and address the social determinants of health that contribute to Indigenous health inequities.

## 5 Strengths and limitations

To the best of our knowledge, this study is the first to systematically examine Indigenous health and wellbeing research using scientometric analysis and knowledge mapping - filling a significant gap in the existing literature. We review a substantial body of literature in a precise and objective manner, to provide insights for researchers engaged in the field. An exploration of the interrelationships among authors, institutions, countries/regions, journals, keywords, citations/co-citations, and references strengthens the robustness of our findings. Nonetheless, it is crucial to acknowledge the existence of limitations.

Firstly, we selected the WoSCC database as a comprehensive and respected platform for bibliometric analysis. Despite the standardization and consistency of publication records, there remains the potential that this approach is a non-exhaustive exploration of the literature (Kulkarni et al., 2009). Second, the influence of newly published articles might be undervalued due to their limited time for citation accrual. We acknowledge that document frequency is not a catalyst for change alone, so to mitigate this, a qualitative synthesis was applied in the analysis and interpretation of the results. Lastly, because software is used to conduct the analysis, there is a potential for errors or biases in our findings. For instance, journal names or research categorization might have changed over time, and there may be instances where two authors sharing the same name are repeatedly aggregated. All efforts to avoid these instances were applied before data analysis.

Despite not being within the scope of this study, we still acknowledge that distinguishing between research *on* Indigenous peoples and research *with* Indigenous peoples is critical. The latter necessitates a sense of relational accountability and research solely *on* Indigenous peoples often produces findings with diminished validity and reliability, and in the worst cases, it exacerbates the persistent overrepresentation of Indigenous populations facing significant challenges to their wellbeing.

## 6 Conclusion

This study represents a comprehensive scientometric analysis and knowledge mapping of Indigenous health and wellbeing research in Australia spanning 2003 to 2022. Our findings not only highlight a substantial and escalating focus of research within this field but also a change from population-level and data-driven studies towards community-based practices and applied research methodologies. Looking ahead in Indigenous health and wellbeing research, we can anticipate a growing emphasis on practices and methodologies that give precedence to forging robust partnerships *with* Indigenous communities. This shift away from conventional deficit mindsets, coupled with a heightened focus on recognizing cultural protocols and privacy considerations, will increasingly underpin the exploration of Indigenous individuals’ experiences as they navigate their health and wellbeing. In an era of heightened awareness and significant investment in Indigenous health and wellbeing research in Australia, the imperative to articulate and prioritize outcomes for Indigenous peoples is more compelling than ever. By presenting this updated perspective based on two decades of published literature, this study not only provides an enhanced understanding of the knowledge in this field but also guides future research efforts.

## Data availability statement

The original contributions presented in the study are included in the article/Supplementary material, further inquiries can be directed to the corresponding author.

## Author contributions

MK: Conceptualization, data Curation, Formal Analysis, Investigation, Methodology. Visualisation, Writing – original draft, Writing – review & editing. KH: Conceptualization, Investigation, Writing – original draft, Writing – review & editing. PA: Conceptualization, Investigation, Writing – original draft, Writing – review & editing. CS: Investigation, Methodology, Resources, Writing – review & editing, Writing – original draft.

## Footnotes

^1^To include all Aboriginal and Torres Strait Islander peoples, while recognizing that Aboriginal and Torres Strait Islander peoples have distinct cultures and identities.
