# Supplementary material for: Mapping the knowledge structure and trends in Australian Indigenous health and wellbeing research from 2003 to 2022: a scientometric analysis
Source: Front Sociol. 2023 Nov 30;8:1290322. doi: 10.3389/fsoc.2023.1290322 (PMC10720666; doi:10.3389/fsoc.2023.1290322)
Supplement: Supplementary file 1 [file Table_1.docx]

Supplementary Material

Mapping the knowledge structure and trends in Australian Indigenous health and wellbeing research from 2003 to 2022: a scientometric analysis

**Michelle A. Krahe^1,2*^, Kerry K. Hall^1^, Peter J. Anderson^1^, Cindy Shannon^1^**

**^1^** Office of the Deputy Vice Chancellor (Indigenous, Diversity and Inclusion), Griffith University, Meadowbrook, QLD, Australia

^2^College of Medicine and Dentistry, James Cook University, Cairns, QLD, Australia*** Correspondence:** Michelle A. Krahe: michelle.krahe@jcu.edu.au

# Supplementary Tables

**Supplementary File 1.** Major clusters of co-cited documents

| **Cluster ID** | **Cluster size** | **Silhouette** | **Label** | **Top Terms (log-likelihood ratio, p-level)** |
| --- | --- | --- | --- | --- |
| #0 | 74 | 0.799 | disadvantage | disadvantage (6.61, 0.05); indigenous/aboriginal health (5.13, 0.05); j15 (5.13, 0.05); perinatal (5.13, 0.05); public good (5.13, 0.05) |
| #1 | 60 | 0.981 | first nations | first nations (19.55, 1.0E-4); wellbeing (12.82, 0.001); global health (7.79, 0.01); program evaluation (7.79, 0.01); young people (7.79, 0.01) |
| #2 | 57 | 0.913 | child health | child health (10.71, 0.005); suicide (10.71, 0.005); psychological distress (10.71, 0.005); indigenous population (8.6, 0.005); nutrition (8.03, 0.005) |
| #3 | 47 | 0.898 | cancer | cancer (24.96, 1.0E-4); myocardial infarction (13.11, 0.001); ischaemic heart disease (13.11, 0.001); treatment (8.85, 0.005); palliative care (8.73, 0.005) |
| #4 | 33 | 0.989 | renal disease | renal disease (25.87, 1.0E-4); transplantation (8.52, 0.005); indigenous renal disease (8.52, 0.005); biological pathways (8.52, 0.005); chronic diseases (8.52, 0.005) |
| #5 | 33 | 0.95 | social determinants | social determinants (13.82, 0.001); maternal (9.2, 0.005); australian (7.13, 0.01); remote health (4.59, 0.05); culturally responsive (4.59, 0.05) |
| #6 | 23 | 1 | substance abuse | cannabis (24.36, 1.0E-4); indigenous australians (10.17, 0.005); arnhem land (9.46, 0.005); substance abuse (9.46, 0.005); petrol sniffing (9.46, 0.005) |
| #7 | 16 | 0.918 | community-based | community-based (7.36, 0.01); world health organisation (7.36, 0.01); ethnicity (7.36, 0.01); postcolonial (7.36, 0.01); hearing (7.36, 0.01) |
| #8 | 12 | 0.981 | epidemiology | epidemiology (15.02, 0.001); trachoma (10.16, 0.005); refractive error (10.16, 0.005); associations (10.16, 0.005); cataract (10.16, 0.005) |
| #9 | 11 | 0.955 | children | children (13.05, 0.001); oral health (9.78, 0.005); rural and remote (9.78, 0.005); location (9.78, 0.005); social inequalities (9.78, 0.005) |

**Supplementary File 2.** Major clusters of co-occurring keywords

| **Cluster ID** | **Cluster size** | **Silhouette** | **Label** | **Top Terms (log-likelihood ratio, p-level)** |
| --- | --- | --- | --- | --- |
| #0 | 35 | 0.634 | cultural safety | cultural safety (21.24, 1.0E-4); care (17.02, 1.0E-4); aboriginal health (16.78, 1.0E-4); rehabilitation (15.23, 1.0E-4); mortality (15.08, 0.001) |
| #1 | 29 | 0.599 | mental health | mental health (43.72, 1.0E-4); public health (35.75, 1.0E-4); health policy (13.54, 0.001); qualitative research (12.8, 0.001); social determinants (11.84, 0.001) |
| #2 | 29 | 0.643 | northern territory | northern territory (23.62, 1.0E-4); indigenous australians (17.59, 1.0E-4); cannabis (15.75, 1.0E-4); mortality (10.38, 0.005); primary health care (9.65, 0.005) |
| #3 | 22 | 0.779 | disease | disease (18.34, 1.0E-4); children (16.28, 1.0E-4); infants (15.56, 1.0E-4); infections (15.56, 1.0E-4); indigenous health (13.7, 0.001) |
| #4 | 22 | 0.755 | indigenous health | indigenous health (51.09, 1.0E-4); primary health care (28.91, 1.0E-4); risk factors (15.52, 1.0E-4); cardiovascular disease (14.29, 0.001); western australia (11.02, 0.001) |

**Supplementary File 3**. Top 10 highly cited documents in Indigenous health and wellbeing (2003 -2022)

| **Rank** | **Authors** | **Year** | **Title** | **Journal** | **Citations** |
| --- | --- | --- | --- | --- | --- |
| 1 | Anderson I, et al. | 2016 | Indigenous and tribal peoples' health (The Lancet-Lowitja Institute Global Collaboration): a population study | Lancet | 481 |
| 2 | Vos T, et al. | 2009 | Burden of disease and injury in Aboriginal and Torres Strait Islander peoples: the Indigenous health gap | International Journal of Epidemiology | 389 |
| 3 | Anderson I, et al. | 2006 | Indigenous health in Australia, New Zealand, and the Pacific | Lancet | 217 |
| 4 | Larson A, et al. | 2007 | It's enough to make you sick: the impact of racism on the health of Aboriginal Australians | Australian and New Zealand Journal of Public Health | 194 |
| 5 | McGorry PD, et al. | 2007 | Australia's National Youth Mental Health Foundation - where young minds come first | Medical Journal of Australia | 189 |
| 6 | Fisher JRW, et al. | 2020 | Mental health of people in Australia in the first month of COVID-19 restrictions: a national survey | Medical Journal of Australia | 152 |
| 7 | Cunningham J, et al. | 2008 | Incidence, aetiology, and outcomes of cancer in Indigenous peoples in Australia | Lancet Oncology | 141 |
| 8 | Burgess CP, et al. | 2009 | Healthy country, healthy people: the relationship between Indigenous health status and caring for country | Medical Journal of Australia | 138 |
| 9 | Sherwood J | 2013 | Colonisation - it's bad for your health: the context of Aboriginal health | Contemporary Nurse | 136 |
| 10 | Dockery AM | 2010 | Culture and wellbeing: the case of Indigenous Australians | Social Indicators Research | 136 |
